# Supplementary material for: Disease clusters subsequent to anxiety and stress-related disorders and their genetic determinants
Source: Nat Commun. 2024 Feb 8;15:1209. doi: 10.1038/s41467-024-45445-2 (PMC10853285; doi:10.1038/s41467-024-45445-2)
Supplement: Supplementary file 1 — Supplementary Information [file 41467_2024_45445_MOESM1_ESM.pdf]

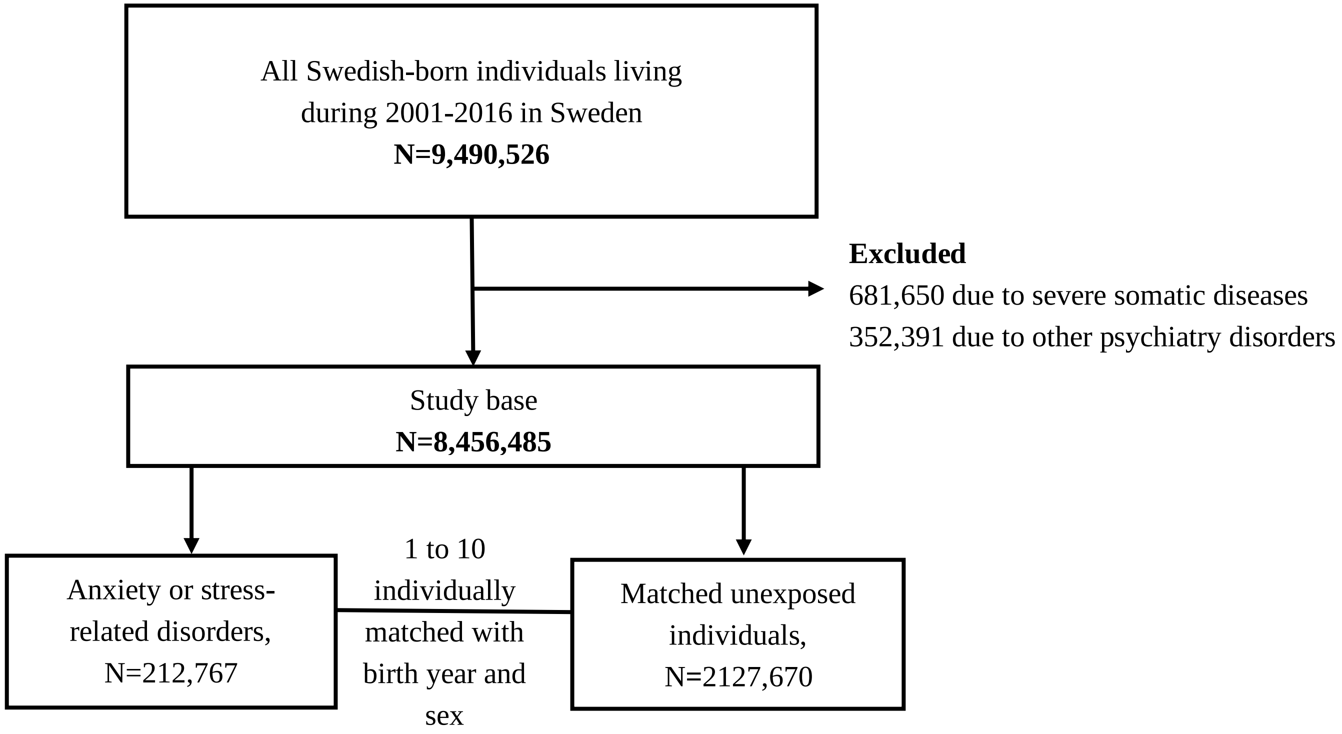


Supplementary Figure 1 Flow chart of the participants selection in the Swedish cohort

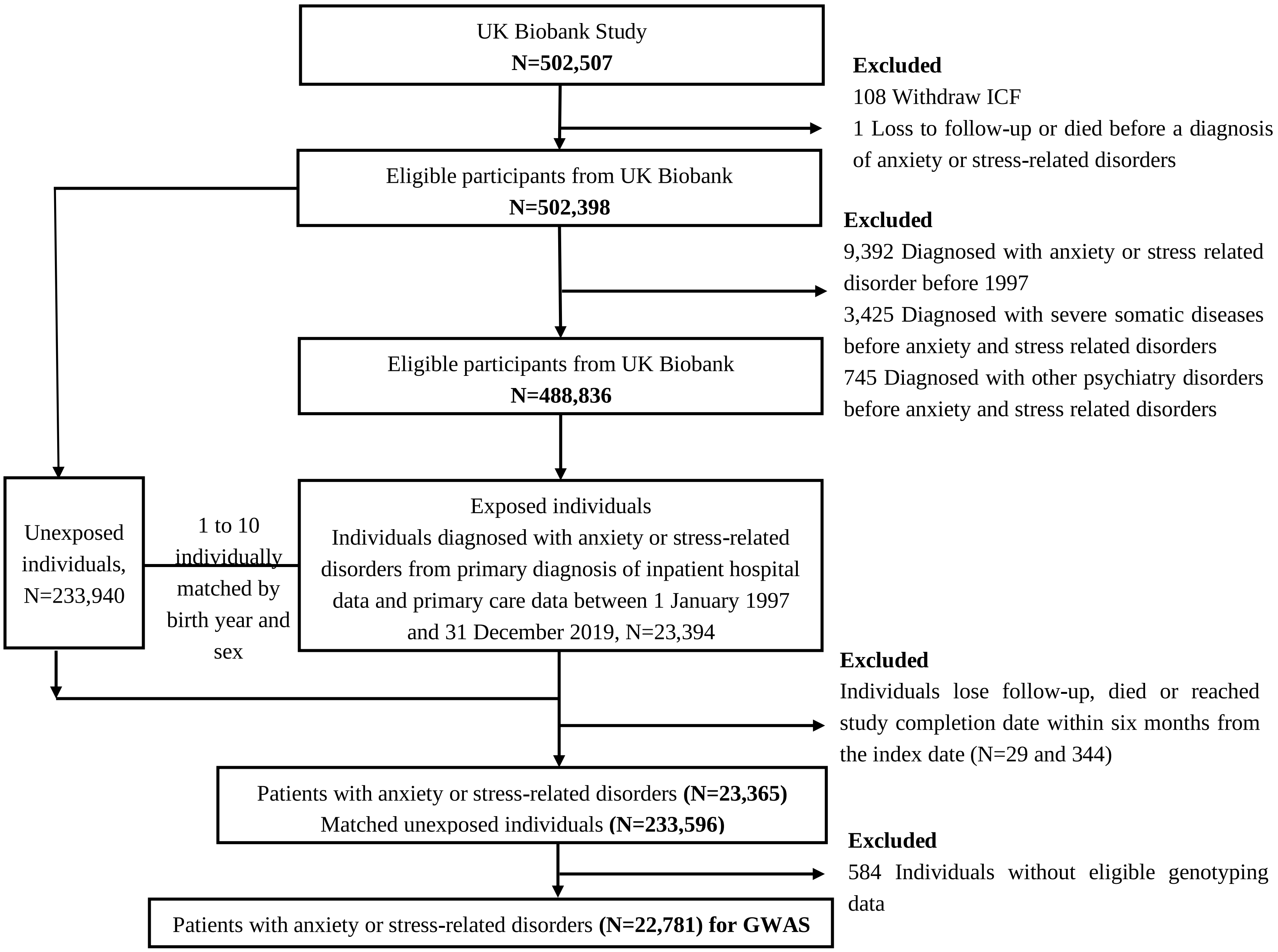


Supplementary Figure 2 Flow chart of the participants selection in the UK cohort

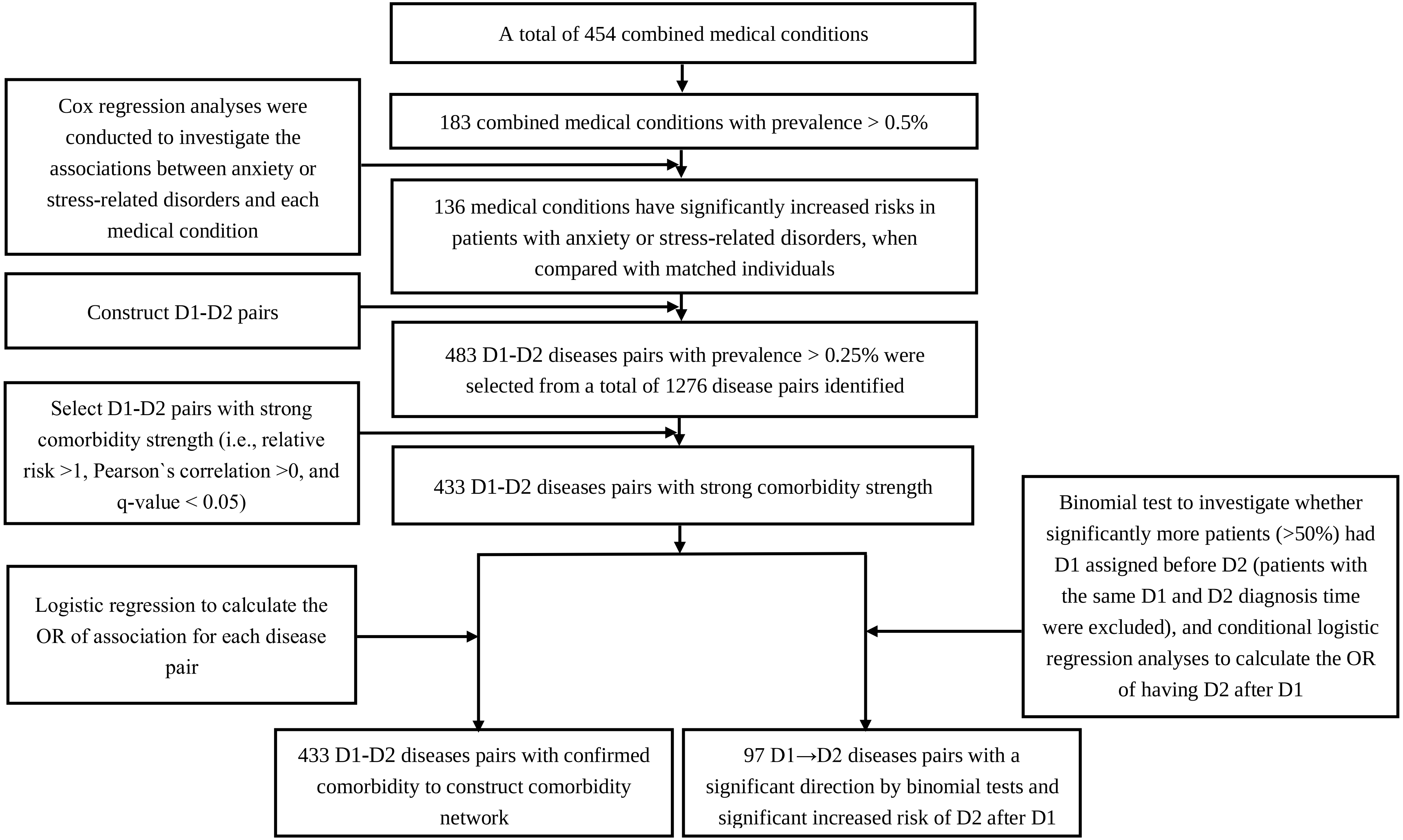


Supplementary Figure 3 Flow chart of identifying disease pairs for comorbidity network and disease trajectories in the Swedish cohort

A Disease clusters after anxiety and stress-related disorders

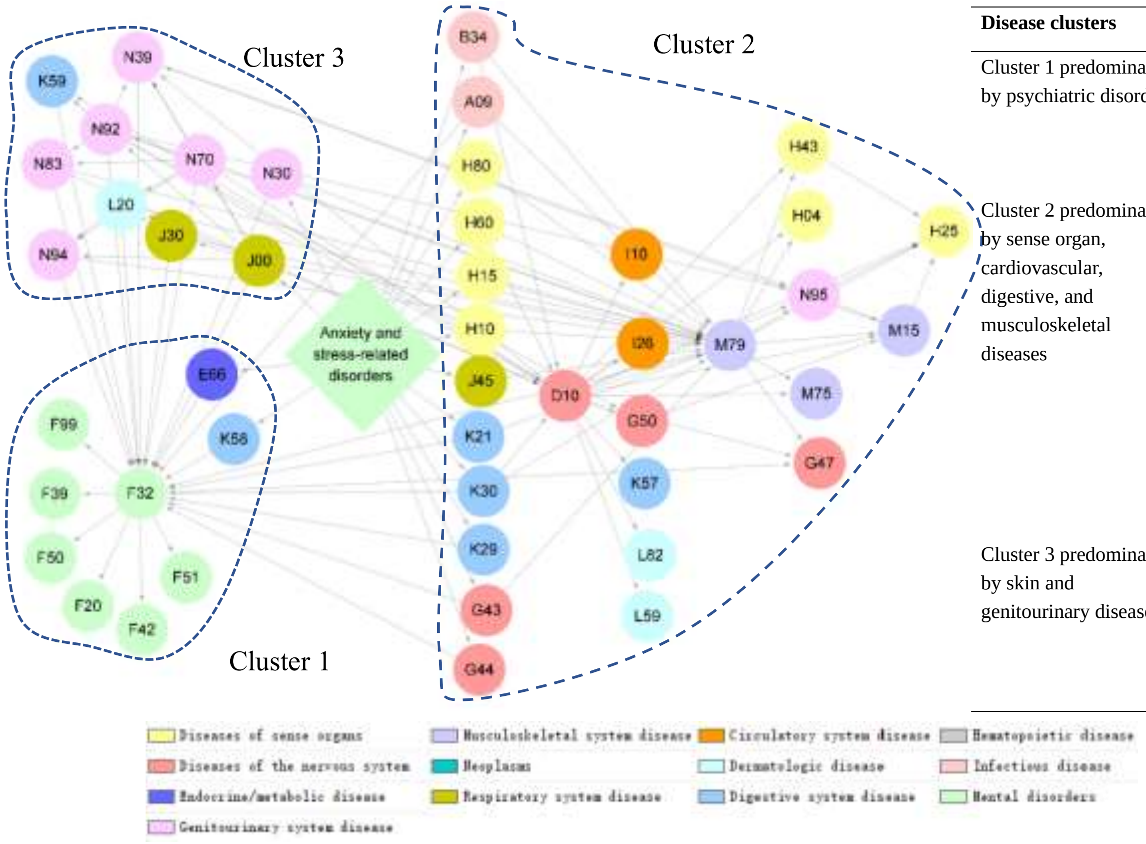


B Combined ICD-10 codes of disease list for each disease cluster after anxiety and stress-related disorders

| **Disease clusters** | **Combined ICD-10 codes (description) of disease list for each disease clusters** |
|---|---|
| Cluster 1 predominated by psychiatric disorders | E66 (Obesity), F20 (Schizophrenia), F32 (Depression), F39 (Other mood disorder), F42 (Obsessive-compulsive disorder), F50 (Eating disorder), F51 (Sleep disorder not due to a sub or known physiological condition), F99 (Mental disorder, not otherwise specified), K58 (Irritable bowel syndrome) |
| Cluster 2 predominated by sense organ, cardiovascular, digestive, and musculoskeletal diseases | A09 (Infectious gastroenteritis and colitis), B34 (Other viral diseases), D10 (Benign tumors), G43 (Migraine), G44 (Other headache syndromes), G47 (Sleep disorder), G50 (Diseases in nerves, nerve roots and nerve plexa), H04 (Disorder of lacrimal system), H10 (Diseases of the conjunctivae), H15 (Diseases of the sclerae, cornea, iris and ciliary body), H25 (Disorders of the lens), H43 (Disorders of the vitreous body and globe), H60 (Infections of the ear), H80 (Diseases of the inner ear), I10 (Hypertensive disorders), I26 (Embolism and thrombosis), J45 (Asthma), K21 (Gastro-esophageal reflux disease), K29 (Gastritis and duodenitis), K30 (Functional dyspepsia), K57 (Diverticular disease of intestine), L59 (Radiation-related disorders of the skin and subcutaneous tissue), L82 (Other seborrheic keratosis), M15 (Osteoarthritis), M75 (Shoulder lesion), M79 (Other soft tissue disorders, not elsewhere classified), N95 (Menopausal and perimenopausal disorders) |
| Cluster 3 predominated by skin and genitourinary diseases | J00 (Upper respiratory infections), J30 (Rhinitis), K59 (Other functional intestinal disorders), L20 (Dermatitis), N30 (Cystitis), N39 (Disorders of urinary system, possibly infection), N70 (Inflammatory diseases of the female pelvic organs), N83 (Noninflammatory disorders of ovary, fallopian tube and broad ligament), N92 (Irregular menstruation), N94 (Pain and other conditions associated with female genital organs and menstrual cycle) |

Supplementary Figure 4 Disease clusters and its disease list among all patients with anxiety and stress-related disorders (n=212,767)

A Disease clusters after anxiety and stress-related disorders. Each node represents a medical condition, and the combined ICD-10 codes are shown within the circle, while the

color of the node indicates the category of the corresponding medical condition. The network contains three disease clusters, and nodes belonging to the same disease cluster were grouped together using blue dashes. B Combined ICD-10 codes of disease list for each disease cluster after anxiety and stress-related disorders. This table listed combined ICD-10 codes for each disease cluster. Definition of combined ICD-10 codes can be found in Supplementary Data 1.

A Disease clusters after anxiety disorder

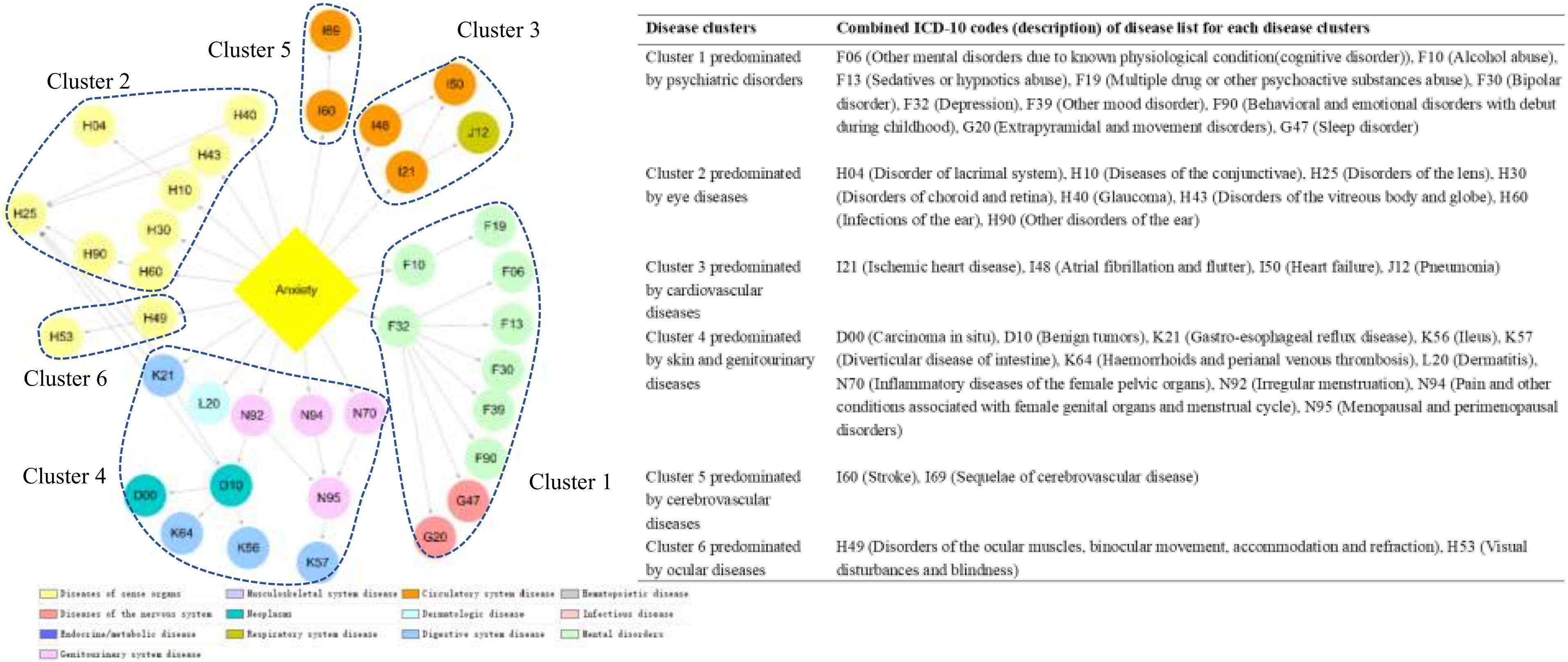


B Combined ICD-10 codes of disease list for each disease clusters after anxiety disorder

| **Disease clusters** | **Combined ICD-10 codes (description) of disease list for each disease clusters** |
|---|---|
| Cluster 1 predominated by psychiatric disorders | F06 (Other mental disorders due to known physiological condition(cognitive disorder)), F10 (Alcohol abuse), F13 (Sedatives or hypnotics abuse), F19 (Multiple drug or other psychoactive substances abuse), F30 (Bipolar disorder), F32 (Depression), F39 (Other mood disorder), F90 (Behavioral and emotional disorders with debut during childhood), G20 (Extrapyramidal and movement disorders), G47 (Sleep disorder) |
| Cluster 2 predominated by eye diseases | H04 (Disorder of lacrimal system), H10 (Diseases of the conjunctivae), H25 (Disorders of the lens), H30 (Disorders of choroid and retina), H40 (Glaucoma), H43 (Disorders of the vitreous body and globe), H60 (Infections of the ear), H90 (Other disorders of the ear) |
| Cluster 3 predominated by cardiovascular diseases | I21 (Ischemic heart disease), I48 (Atrial fibrillation and flutter), I50 (Heart failure), J12 (Pneumonia) |
| Cluster 4 predominated by skin and genitourinary diseases | D00 (Carcinoma in situ), D10 (Benign tumors), K21 (Gastro-esophageal reflux disease), K56 (Ileus), K57 (Diverticular disease of intestine), K64 (Haemorrhoids and perianal venous thrombosis), L20 (Dermatitis), N70 (Inflammatory diseases of the female pelvic organs), N92 (Irregular menstruation), N94 (Pain and other conditions associated with female genital organs and menstrual cycle), N95 (Menopausal and perimenopausal disorders) |
| Cluster 5 predominated by cerebrovascular diseases | I60 (Stroke), I69 (Sequelae of cerebrovascular disease) |
| Cluster 6 predominated by ocular diseases | H49 (Disorders of the ocular muscles, binocular movement, accommodation and refraction), H53 (Visual disturbances and blindness) |

Supplementary Figure 5 Disease clusters and its disease list among patients with anxiety disorder (n=39,322)

A Disease clusters after anxiety and stress-related disorders. Each node represents a medical condition, and the combined ICD-10 codes are shown within the circle, while the color of the node indicates the category of the corresponding medical condition. The network contains six disease clusters, and nodes belonging to the same disease cluster were grouped together using blue dashes. B Combined ICD-10 codes of disease list for each disease cluster after anxiety and stress-related disorders. This table listed combined ICD-10 codes for each disease cluster. Definition of combined ICD-10 codes can be found in Supplementary Data 1.

A Disease clusters after stress-related disorder

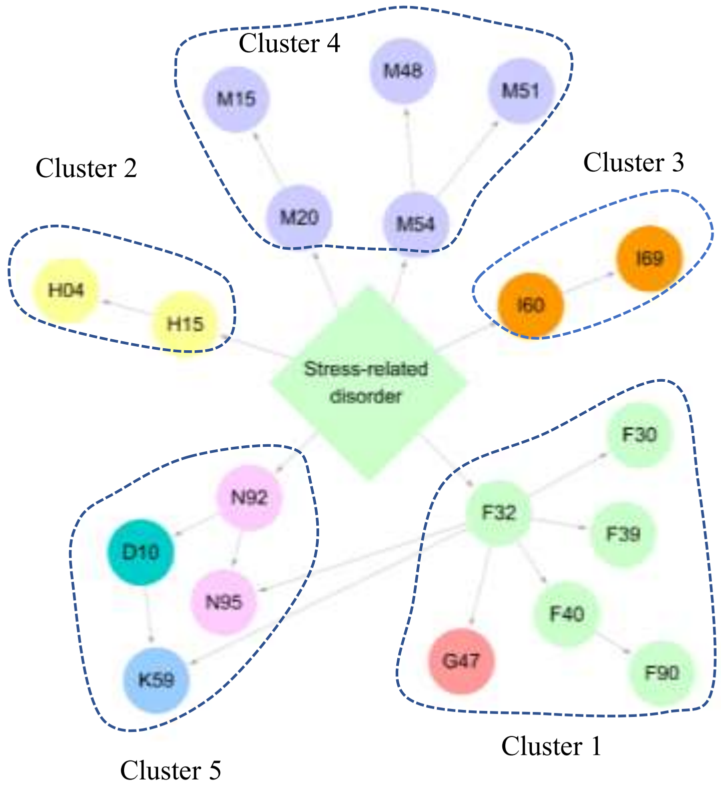


B Combined ICD-10 codes of disease list for each disease clusters after stress-related disorder

| Disease clusters | Combined ICD-10 codes (description) of disease list for each disease clusters |
|---|---|
| Cluster 1 predominated by psychiatric disorders | F30 (Bipolar disorder), F32 (Depression), F39 (Other mood disorder), F40 (Anxiety), F90 (Behavioral and emotional disorders with debut during childhood), G47 (Sleep disorder) |
| Cluster 2 predominated by eye diseases | H04 (Disorder of lacrimal system), H15 (Diseases of the sclerae, cornea, iris and ciliary body) |
| Cluster 3 predominated by cerebrovascular diseases | I60 (Stroke), I69 (Sequelae of cerebrovascular disease) |
| Cluster 4 predominated by musculoskeletal diseases | M15 (Osteoarthritis), M20 (Other joint disorders), M48 ( Spondylopathy), M51(Other intervertebral disc disorders), M54 (Dorsalgia) |
| Cluster 5 predominated by genitourinary diseases | D10 (Benign tumors), K59 (Other functional intestinal disorders), N92 (Irregular menstruation), N95 (Menopausal and perimenopausal disorders) |

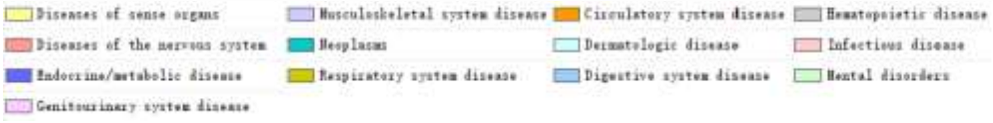


Supplementary Figure 6 Disease clusters and its disease list among patients with stress-related disorder (n=30,704)

A Disease clusters after anxiety and stress-related disorders. Each node represents a medical condition, and the combined ICD-10 codes are shown within the circle, while the color of the node indicates the category of the corresponding medical condition. The network contains five disease clusters, and nodes belonging to the same disease cluster were grouped together using blue dashes. B Combined ICD-10 codes of disease list for each disease cluster after anxiety and stress-related disorders. This table listed combined ICD-10 codes for each disease cluster. Definition of combined ICD-10 codes can be found in Supplementary Data 1.

A Disease clusters after anxiety and stress-related disorders

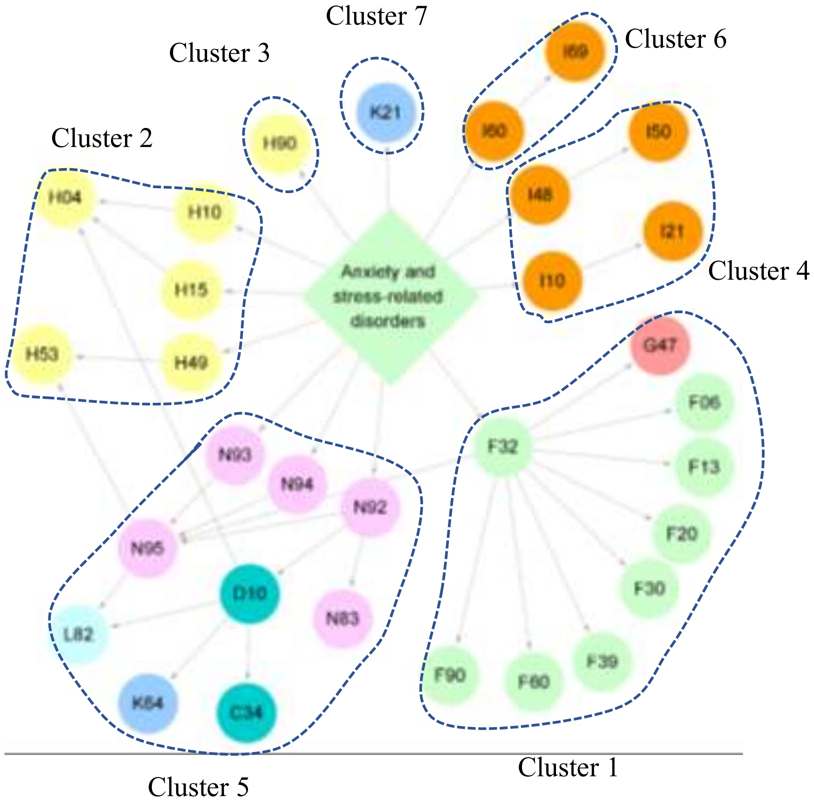


B Combined ICD-10 codes of disease list for each disease cluster after anxiety and stress-related disorders

| Disease clusters | Combined ICD-10 codes (description) of disease list for each disease clusters |
|---|---|
| Cluster 1 predominated by psychiatric disorders | F06(Other mental disorders due to known physiological condition(cognitive disorder)), F13 (Sedatives or hypnotics abuse), F20 (Schizophrenia), F30 (Bipolar disorder), F32 (Depression), F39 (Other mood disorder), F60 (Personality and behavioral disorder), F90 (Behavioral and emotional disorders with debut during childhood), G47 (Sleep disorder) |
| Cluster 2 predominated by eye diseases | H04 (Disorder of lacrimal system), H10 (Diseases of the conjunctivae), H15 (Diseases of the sclerae, cornea, iris and ciliary body), H49 (Disorders of the ocular muscles, binocular movement, accommodation and refraction), H53 (Visual disturbances and blindness) |
| Cluster 3 predominated by ear diseases | H90 (Other disorders of the ear) |
| Cluster 4 predominated by cardiovascular diseases | I10 (Hypertensive disorders), I21 (Ischemic heart disease), I48 (Atrial fibrillation and flutter), I50 (Heart failure) |
| Cluster 5 predominated by skin and genitourinary diseases | C34 (Lung and bronchus cancer), D10 (Benign tumors), K64 (Haemorrhoids and perianal venous thrombosis), L82 (Other seborrheic keratosis), N83 (Noninflammatory disorders of ovary, fallopian tube and broad ligament), N92 (Irregular menstruation), N93 (Other abnormal uterine and vaginal bleeding), N94 (Pain and other conditions associated with female genital organs and menstrual cycle), N95 (Menopausal and perimenopausal disorders) |
| Cluster 6 predominated by cerebrovascular diseases | I60 (Stroke), I69 (Sequelae of cerebrovascular disease) |
| Cluster 7 predominated by digestive diseases | K21 (Gastro-esophageal reflux disease) |

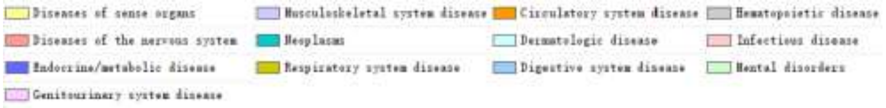


Supplementary Figure 7 Disease clusters and its disease list among female patients with anxiety and stress-related disorders (n=43,933)

A Disease clusters after anxiety and stress-related disorders. Each node represents a medical condition, and the combined ICD-10 codes are shown within the circle, while the color of the node indicates the category of the corresponding medical condition. The network contains seven disease clusters, and nodes belonging to the same disease cluster

were grouped together using blue dashes. B Combined ICD-10 codes of disease list for each disease cluster after anxiety and stress-related disorders. This table listed combined ICD-10 codes for each disease cluster. Definition of combined ICD-10 codes can be found in Supplementary Data 1.

A Disease clusters after anxiety and stress-related disorders

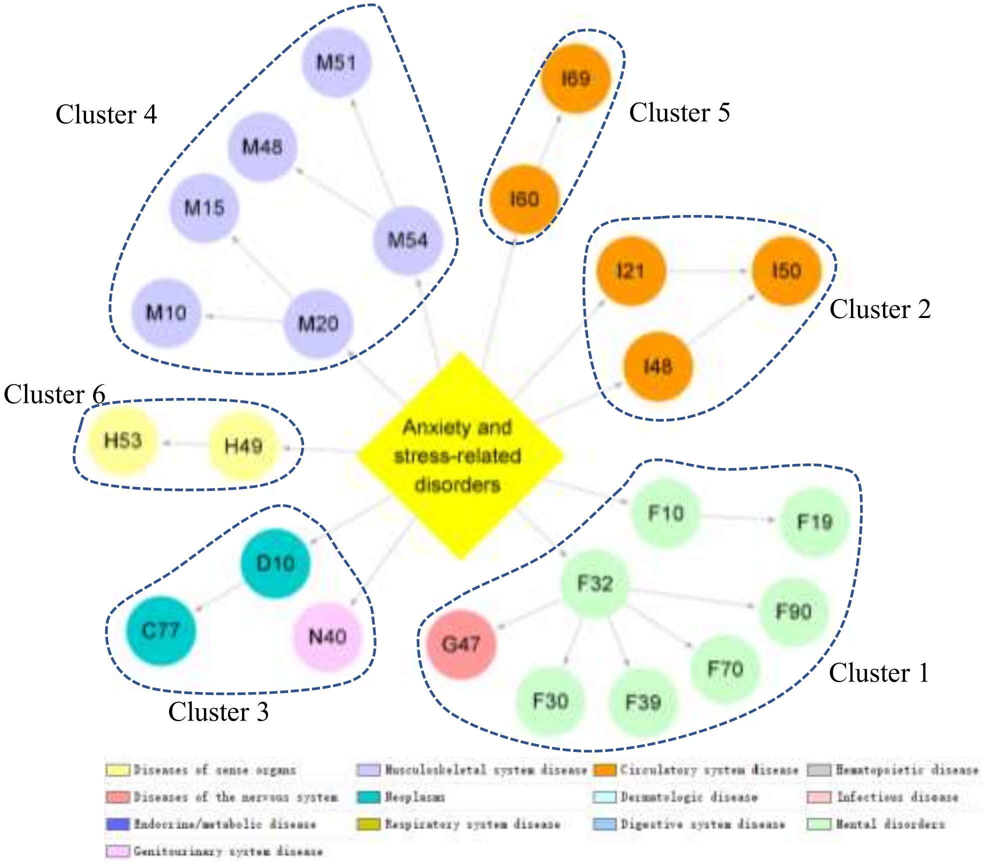


B Combined ICD-10 codes of disease list for each disease clusters after anxiety and stress-related disorders

| **Disease clusters** | **Combined ICD-10 codes (description) of disease list for each disease clusters** |
|---|---|
| Cluster 1 predominated by psychiatric disorders | F10 (Alcohol abuse), F19 (Multiple drug or other psychoactive substances abuse), F30 (Bipolar disorder), F32 (Depression), F39 (Other mood disorder), F70 (Mental retardation), F90 (Behavioral and emotional disorders with debut during childhood), G47 (Sleep disorder) |
| Cluster 2 predominated by cardiovascular diseases | I21 (Ischemic heart disease), I48 (Atrial fibrillation and flutter), I50 (Heart failure) |
| Cluster 3 predominated by neoplasm and genitourinary diseases | C77 (Metastatic cancer), D10 (Benign tumors), N40 (Hyperplasia of prostate) |
| Cluster 4 predominated by musculoskeletal diseases | M10 (Other inflammatory arthritis), M15 (Osteoarthritis), M20 (Other joint disorders), M48 (Spondylopathy), M51 (Other intervertebral disc disorders), M54 (Dorsalgia) |
| Cluster 5 predominated by cerebrovascular diseases | I60 (Stroke), I69 (Sequelae of cerebrovascular disease) |
| Cluster 6 predominated by ocular diseases | H49 (Disorders of the ocular muscles, binocular movement, accommodation and refraction), H53 (Visual disturbances and blindness) |

Supplementary Figure 8 Disease clusters and its disease list among male patients with anxiety and stress-related disorders (n=26,093)

A Disease clusters after anxiety and stress-related disorders. Each node represents a medical condition, and the combined ICD-10 codes are shown within the circle, while the color of the node indicates the category of the corresponding medical condition. The network contains six disease clusters, and nodes belonging to the same disease cluster

were grouped together using blue dashes. B Combined ICD-10 codes of disease list for each disease cluster after anxiety and stress-related disorders. This table listed combined ICD-10 codes for each disease cluster. Definition of combined ICD-10 codes can be found in Supplementary Table 1.

Supplementary Table 1 Disease clusters and its disease list among individuals with anxiety and stress-related disorders validated in the UK cohort

| Disease clusters | Combined ICD-10 codes of disease list for each disease clusters in SNR (N=70,026) | Combined ICD-10 codes of disease list for each disease clusters in UKB (N=23,365) |
|---|---|---|
| Cluster 1 predominated by psychiatric disorders# | E66, F10, F13, F19, F20, F30, F32, F39, F60, F90 | E66, F10, F30, F32 |
| Cluster 2 predominated by eye diseases | H04, H10, H15, H25, H40, H49, H53 | H25, H49, H53 |
| Cluster 3 predominated by ear diseases# | H60, H90 | H90 |
| Cluster 4 predominated by cardiovascular diseases | I10, I20, I21, I50, J12 | I20, I21 |
| Cluster 5 predominated by skin and genitourinary diseases | L20, L40, L82, N39, N92, N94, N95 | N92, N94 |

SNR: Swedish national registers; UKB: UK Biobank.

*Combined ICD-10 code; mapping between the original ICD-10 code and the combined ICD-10 code can be found in the Supplementary Table 1;

#These two clusters were merged into one cluster.

Supplementary Table 2 LD score regression of GWAS for each disease cluster subsequent to anxiety and stress-related disorders

| Disease clusters | $\lambda_{GC}$ | Intercept (SE) | Attenuation ratio |
|---|---|---|---|
| Cluster 1 predominated by psychiatric disorders | 1.0225 | 0.9914 (0.0062) | < 0 |
| Cluster 2 predominated by eye diseases | 1.0105 | 0.9987 (0.0058) | < 0 |
| Cluster 3 predominated by ear diseases | 1.0007 | 1.0023 (0.0073) | 3.1828 |
| Cluster 4 predominated by cardiovascular diseases | 1.0225 | 0.9827 (0.0075) | < 0 |
| Cluster 5 predominated by skin and genitourinary diseases | 1.0135 | 0.9951 (0.0058) | < 0 |

$\lambda_{GC}$: genomic control inflation factor; SE: standard error

Supplementary Table 3 Baseline characteristics of all participants from the Swedish cohort

| | Individuals with anxiety and stress-related disorders (N= 212,767) | Matched individuals (N= 2,127,670)* |
|---|---|---|
| Age at diagnosis in years, median (IQR) | 32 (22-45) | 32 (22-45) |
| Follow-up time in years, median (IQR) | 6.7 (3.1-10.4) | 6.7 (3.2-10.5) |
| Sex (%) | | |
| Male | 79,801 (37.5) | 798,010 (37.5) |
| Female | 132,966 (62.5) | 1,329,660 (62.5) |
| Highest Education (%) | | |
| <9 yr | 47,177 (22.2) | 335,003 (15.7) |
| 9-12 yr | 97,813 (46.0) | 944,300 (44.4) |
| >12 yr | 67,777 (31.9) | 848,367 (39.9) |
| Income (%) | | |
| Lowest 20% | 45,864 (21.6) | 422,072 (19.8) |
| Middle | 135,230 (63.6) | 1,267,925 (59.6) |
| Highest 20% | 31,634 (14.9) | 435,951 (20.5) |
| Missing | 39 (0.02) | 1,722 (0.08) |

Abbreviation: IRQ: interquartile range.

* At most 10 participants who were alive, retained in the cohort and free of anxiety and stress-related disorders at the corresponding index date were individually matched to each individual with anxiety and stress-related disorders based on sex, and year of birth.

Supplementary Table 4 Primary care codes for diseases identifications in the UK cohort

| | Primary care READ V2 code | Primary care READ V3 code |
|---|---|---|
| Anxiety disorder | E2000, E2001, E2002, E2004, E2005, E200z, E202., E2020, E2021, E2022, E2023, E2024, E2025, E2026, E2027, E2028, E2029, E202A,E202B, E202C, E202D, E202E, E202z, Eu40., Eu400, Eu401, Eu402, Eu403, Eu40y, Eu40z, Eu41., Eu410, Eu411, Eu412, Eu413, Eu41y, Eu41z | E200., E2000, E2002, E2004, E200z, E2020, E2021, E2022, E2023, E2024, E2025, E2026, E2027, E2028, E2029, E202E, Eu40., Eu400, Eu402, Eu40y, Eu40z, Eu41., Eu410, Eu413, Eu41y, Eu41z, Eu931, Ua1qa, Ua1qc, Ua1qd, Ua1qe, Ua1qf, Ua1qg, Ua1qh, Ua1qi, Ua1qj, Ua1qk, Ua1ql, , Ua1qm, Ua1qn, Ua1qo, Ua1qp, Ua1qs, Ua1qt, Ua1qU, Ua1qV, Ua1qW, Ua1qX, Ua1qY, X00RP, X00Sa, X00Sb, X00Sc, X00Sd, X00Se, X00Sr, X00SV, X00SW, X00SX, X00SY, X00SZ, X50G2, X50G3, X50G5, X50G6, X50GI, X75YV, X761d, X761n, X761q, X761t, X761u, X761y, X7627, X7628, X7629, X762a, X762C, X762E, X762F, X762G, X762H, X762T, X762Z, X78wp, Xa00r, Xa00s, Xa1a8, Xa1Ev, Xa3Vj, Xa3Vk, Xa3Vl, Xa3WH, Xa3WI, Xa3WJ, Xa7k9, Xa7kB, XaIo7, XaKVA, XE0rb, XE1Y7, XE1YA, XE1YB, XE1Zj, XM0Ak |
| Stress-related disorder | E280., E281., E282., E283., E2830, E2831, E283z, E284., E28z., E2900, E2925, E292y, E292z, E293., E2930, E2931, E2932, E293z, E294., E29y., E29y1, E29y2, E29y4, E29yz, E29z., Eu43., Eu430, Eu431, Eu432, Eu433, Eu434, Eu435, Eu43y, Eu43z | 1B1L., 1BE.., E280., E281., E282., E283., E2830, E2831, E283z, E284., E29.., E290., E290z, E291., E292., E2920, E2921, E2922, E2924, E2925, E292y, E292z, E293., E2930, E2931, E2932, E293z, E294., E29y., E29y0, E29y1, E29y2, E29y3, E29y4, E29y5, E29yz, E29z., Eu430, Eu432, Eu43y, Eu43z, Eu930, Ry15., Ua18k, Ua18L, Ub1T9, X00Sf, X00TT, X40Js, Xa028, Xa18j, Xa18v, XaC2u, XaX55, XaX56, XaX58, XE1Ym, XE1Yn, XE1Yo, XE1Yp, XE2uz, XM0As, XM1Q3 |
