# Supplementary material for: Disease clusters subsequent to anxiety and stress-related disorders and their genetic determinants
Source: Nat Commun. 2024 Feb 8;15:1209. doi: 10.1038/s41467-024-45445-2 (PMC10853285; doi:10.1038/s41467-024-45445-2)
Supplement: Supplementary file 3 — Description of Additional Supplementary Files [file 41467_2024_45445_MOESM3_ESM.pdf]

Description of Additional Supplementary Files

File Name: Supplementary Data 1
Description: Identification of Combined ICD-10 codes.

File Name: Supplementary Data 2
Description: PheWAS results for anxiety and stress-related disorders in the Swedish cohort.

File Name: Supplementary Data 3
Description: Odds ratios (ORs) with 95% confidence intervals (CIs) for the significant disease pairs after anxiety and stress-related disorders validated in the comorbidity analysis in the Swedish cohort.

File Name: Supplementary Data 4
Description: Odds ratios (ORs) with 95% confidence intervals (CIs) for the significant disease pairs after anxiety and stress-related disorders validated in the trajectory analysis in the Swedish cohort.

File Name: Supplementary Data 5
Description: Hazard ratios (HRs) with 95% confidence intervals (CIs) for the significant medical conditions after anxiety and stress-related disorders validated in the UK cohort.

File Name: Supplementary Data 6
Description: Odds ratios (ORs) with 95% confidence intervals (CIs) for the significant disease pairs after anxiety and stress-related disorders validated in the UK cohort.

File Name: Supplementary Data 7
Description: Lists of SNPs for disease Clusters associated with anxiety and stress-related disorders in the Swedish cohort.

File Name: Supplementary Data 8
Description: Lists of mapped genes for disease clusters associated with anxiety and stress-related disorders in the Swedish cohort.

File Name: Supplementary Data 9
Description: Top 20 enrichment biological pathways for disease clusters associated with anxiety and stress-related disorders in the Swedish cohort.

File Name: Supplementary Data 10
Description: Mapped genes and their reported cluster-related traits from FUMA and GeneCards.

File Name: Supplementary Data 11
Description: Genetic determinants for five disease clusters associated with anxiety and stress-related disorders in the UK cohort.

File Name: Supplementary Data 12
Description: Number of mapped genes in GWAS of disease clusters among individuals with/without anxiety/stress-related disorders.
