# Supplementary material for: Disease clusters subsequent to anxiety and stress-related disorders and their genetic determinants
Source: Nat Commun. 2024 Feb 8;15:1209. doi: 10.1038/s41467-024-45445-2 (PMC10853285; doi:10.1038/s41467-024-45445-2)
Supplement: Supplementary file 5 — Reporting Summary [file 41467_2024_45445_MOESM5_ESM.pdf]

nature portfolio

Corresponding author(s): Huan Song

Last updated by author(s): Jan 9, 2024

Reporting Summary

Nature Portfolio wishes to improve the reproducibility of the work that we publish. This form provides structure for consistency and transparency in reporting. For further information on Nature Portfolio policies, see our Editorial Policies and the Editorial Policy Checklist.

Statistics

For all statistical analyses, confirm that the following items are present in the figure legend, table legend, main text, or Methods section.

n/a	Confirmed	
☐	☒	The exact sample size (n) for each experimental group/condition, given as a discrete number and unit of measurement
☐	☒	A statement on whether measurements were taken from distinct samples or whether the same sample was measured repeatedly
☐	☒	The statistical test(s) used AND whether they are one- or two-sided<br>*Only common tests should be described solely by name; describe more complex techniques in the Methods section.*
☐	☒	A description of all covariates tested
☐	☒	A description of any assumptions or corrections, such as tests of normality and adjustment for multiple comparisons
☐	☒	A full description of the statistical parameters including central tendency (e.g. means) or other basic estimates (e.g. regression coefficient) AND variation (e.g. standard deviation) or associated estimates of uncertainty (e.g. confidence intervals)
☐	☒	For null hypothesis testing, the test statistic (e.g. F, t, r) with confidence intervals, effect sizes, degrees of freedom and P value noted<br>*Give P values as exact values whenever suitable.*
☒	☐	For Bayesian analysis, information on the choice of priors and Markov chain Monte Carlo settings
☐	☒	For hierarchical and complex designs, identification of the appropriate level for tests and full reporting of outcomes
☐	☒	Estimates of effect sizes (e.g. Cohen's d, Pearson's r), indicating how they were calculated

Our web collection on statistics for biologists contains articles on many of the points above.

Software and code

Policy information about availability of computer code

Data collection	No software was used for data collection.
Data analysis	The phenotypic analyses were conducted using SAS 9.4 (SAS Institute), R (Version 4.0.2), Python (Version 3.8) and Cytoscape (Version 3.8.0). PLINK (Version 1.9) and GCTA (Version 1.24) were used for genetic analyses.

For manuscripts utilizing custom algorithms or software that are central to the research but not yet described in published literature, software must be made available to editors and reviewers. We strongly encourage code deposition in a community repository (e.g. GitHub). See the Nature Portfolio guidelines for submitting code & software for further information.

Data

Policy information about availability of data

All manuscripts must include a data availability statement. This statement should provide the following information, where applicable:

- Accession codes, unique identifiers, or web links for publicly available datasets
- A description of any restrictions on data availability
- For clinical datasets or third party data, please ensure that the statement adheres to our policy

The data from Swedish registers are not publicly available due to Swedish law. Data from the UK Biobank (http://www.ukbiobank.ac.uk/) are available to all researchers upon making an application. Part of this research was conducted using the UK Biobank Resource under Application 54803. Other data or platforms are available to all researchers: FUMA (http://fuma.ctglab.nl/), Metascape (https://metascape.org/), GeneCards (https://www.genecards.org/).

Research involving human participants, their data, or biological material

Policy information about studies with human participants or human data. See also policy information about sex, gender (identity/presentation), and sexual orientation and race, ethnicity and racism.

Reporting on sex and gender	The Swedish and the UK cohorts were both overrepresented by females (62.7% and 67.3% respectively), with a similar median age at diagnosis for anxiety and stress-related disorders (Table 1).
Reporting on race, ethnicity, or other socially relevant groupings	By cross linkage to the Total Population Register using the unique Swedish personal identification numbers, we included all Swedish-born individuals residing from 2001 to 2016 in Sweden. We focused on Swedish-born individuals in the present study to reduce the heterogeneity in genetic background as well as other sociodemographic factors, including differential health-seeking behaviors.<br>In the genetic analysis, we excluded non-European ancestry individuals, leaving 22,781 patients with anxiety/stress-related disorders for genetic analysis.
Population characteristics	The Swedish and the UK cohorts were both overrepresented by females (62.7% and 67.3% respectively), with a similar median age at diagnosis for anxiety and stress-related disorders (Table 1). The median follow-up time was 7.1 and 13.3 years in the Swedish cohort and UK cohort, respectively. Patients in both cohorts had lower educational and income levels than their matched unaffected individuals.
Recruitment	The Swedish Patient Register includes nearly complete health records of inpatient care since 1987 and outpatient specialist care since 2001 in Sweden. The UK Biobank (UKB) is a community-based cohort study that enrolled half a million participants aged 40 to 69 at recruitment between 2006 and 2010 across England, Scotland, and Wales.
Ethics oversight	This study was approved by the Ethical Vetting Board in Stockholm, Sweden (DNRs 2012/1814-31/4 and 2015/1062-32), the NHS National Research Ethics Service (16/NW/0274), and the Biomedical Research Ethics Committee of West China Hospital (2019-1171).

Note that full information on the approval of the study protocol must also be provided in the manuscript.

Field-specific reporting

Please select the one below that is the best fit for your research. If you are not sure, read the appropriate sections before making your selection.

☒ Life sciences ☐ Behavioural & social sciences ☐ Ecological, evolutionary & environmental sciences

For a reference copy of the document with all sections, see nature.com/documents/nr-reporting-summary-flat.pdf

Life sciences study design

All studies must disclose on these points even when the disclosure is negative.

Sample size	In the phenotypical analysis, we included all patients with anxiety or stress-related disorders as any first specialist care diagnosis in an inpatient or outpatient hospital visit according to the Swedish Patient Register (N=212,767). To facilitate validation between the Swedish cohort and the UK Biobank, we selected a sub-cohort of the Swedish cohort, namely participants with age >second tertile (median age at index date=51, N=70,026). The nationwide approach gives us the maximal sample size we could have in this research setting. The sample size is sufficient as it is large than our previous study with a similar design (PMID 34035478, N=24,130).
Data exclusions	In the prototypical analysis, since our study period was from 2001 to 2016, we excluded participants with any pre-existing psychiatric disorders or history of severe somatic diseases at the time of diagnosis determined by the Charlson Comorbidity Index (CCI) before 2001. The exclusion criteria have been established in our previous study with a similar design (PMID 34035478).<br>In the genetic analysis, we restricted to the GWASs of European-ancestry individuals in the UK Biobank.
Replication	To validate the identified disease clusters in the Swedish cohort, we constructed a UK cohort (i.e., the validation dataset) based on the UK Biobank. These five clusters and their featured diseases in the Swedish cohort are largely validated in the UK Biobank. Given the nature of observational study, one independent replication is adequate.
Randomization	Randomization is not applicable as this is an observational study.
Blinding	Blinding is not applicable as this is an observational study.

Reporting for specific materials, systems and methods

We require information from authors about some types of materials, experimental systems and methods used in many studies. Here, indicate whether each material, system or method listed is relevant to your study. If you are not sure if a list item applies to your research, read the appropriate section before selecting a response.

Materials & experimental systems

n/a	Involved in the study
☒	☐ Antibodies
☒	☐ Eukaryotic cell lines
☒	☐ Palaeontology and archaeology
☒	☐ Animals and other organisms
☒	☐ Clinical data
☒	☐ Dual use research of concern
☒	☐ Plants

Methods

n/a	Involved in the study
☒	☐ ChIP-seq
☒	☐ Flow cytometry
☒	☐ MRI-based neuroimaging

Plants

Seed stocks	*Report on the source of all seed stocks or other plant material used. If applicable, state the seed stock centre and catalogue number. If plant specimens were collected from the field, describe the collection location, date and sampling procedures.*
Novel plant genotypes	*Describe the methods by which all novel plant genotypes were produced. This includes those generated by transgenic approaches, gene editing, chemical/radiation-based mutagenesis and hybridization. For transgenic lines, describe the transformation method, the number of independent lines analyzed and the generation upon which experiments were performed. For gene-edited lines, describe the editor used, the endogenous sequence targeted for editing, the targeting guide RNA sequence (if applicable) and how the editor was applied.*
Authentication	*Describe any authentication procedures for each seed stock used or novel genotype generated. Describe any experiments used to assess the effect of a mutation and, where applicable, how potential secondary effects (e.g. second site T-DNA insertions, mosiacism, off-target gene editing) were examined.*
